# New insights into the transcription factor regulatory networks driving peel coloration under hormone induction analyzed by transcriptomics and metabolomics in tangor ‘Murcot’

**DOI:** 10.3389/fpls.2025.1526733

**Published:** 2025-02-18

**Authors:** Yang Chen, Lei Yang, Shuang Li, Min Wang, Jianjun Yu, Wenqin Bai, Lin Hong

**Affiliations:** ^1^ Biotechnology Research Institute, Chongqing Academy of Agricultural Sciences, Chongqing, China; ^2^ Chongqing Key Laboratory of Adversity Agriculture Research, Chongqing Academy of Agricultural Sciences, Chongqing, China; ^3^ Key Laboratory of Evaluation and Utilization for Special Crops Germplasm Resource in the Southwest Mountains, Ministry of Agriculture and Rural Affairs, Chongqing, China

**Keywords:** gibberellic acid, ethylene, omics, pigment metabolism, transcription factor

## Abstract

**Introduction:**

Fruit color is a crucial quality factor strongly influencing consumer preference for citrus. The coloration of citrus fruit is primarily determined by carotenoids, which produce a range of hues. Gibberellic acid (GA) and ethylene are critical in fruit coloration during the ripening process. Nevertheless, the underlying mechanisms remain poorly understood.

**Methods:**

The present study utilized transcriptomic and metabolomic analyses to investigate the molecular regulatory mechanisms affecting peel pigment metabolism in tangors (*Citrus reticulata* Blanco×*Citrus sinensis* L. Osbeck) following GA and ethephon (ETH) treatments.

**Results and discussion:**

Collectively, our findings indicated that GA inhibits chlorophyll degradation and the accumulation of numerous carotenoids, including five violaxanthin esters (violaxanthin palmitate, violaxanthin myristate–caprate, violaxanthin myristate–laurate, violaxanthin dilaurate, violaxanthin myristate) and two β-cryptoxanthin derivatives (β–cryptoxanthin laurate, β–cryptoxanthin myristate), while ETH promotes these processes. Furthermore, GA inhibited the downregulation of lutein, the predominant carotenoid in immature fruits. Notably, integrated transcriptomic and metabolomic analyses identified 33 transcription factors associated with pigment metabolism. Of these, two novel transcription factors, the ethylene-responsive transcription factor ABR1 and the HD-Zip transcription factor ATHB7, were uncovered through both transcriptomic analysis and weighted gene co-expression network analysis. These two transcription factors positively regulated the colouration process, as validated by transient overexpression assays in tobacco. Taken together, our findings elucidated the global carotenoid changes and transcriptional alterations in regulating citrus peel color under hormone induction, with significant implications for improving citrus production.

## Introduction

Fruit color is a critical quality parameter, as consumers prefer attractive, bright peel colors (Ruiz-Sola and Rodríguez-Concepción, 2012; Yuan et al., 2015). In immature citrus peel, chlorophyll is the predominant pigment, while the yellow and orange hues observed during ripening are primarily due to the accumulation of carotenoids (Lu et al., 2024; Yuan et al., 2015). In certain citrus varieties, such as blood orange (*Citrus sinensis*), the red pulp coloration is attributed to the accumulation of anthocyanins (Huang et al., 2019). As citrus fruits mature, chloroplasts in the peel transition to chromoplasts, resulting in a rapid decline in chlorophyll content and a concomitant increase in carotenoid levels, facilitating the color change from green to orange (Kato et al., 2004; Ríos et al., 2010). Carotenoids are integral to citrus fruits, enhancing flesh coloration and serving as essential dietary antioxidants and precursors to vitamin A, which are crucial for human health (Cabezas-Terán et al., 2023; Concepcion et al., 2018).

The diverse range of color mutants and carotenoid metabolites in citrus fruits makes them ideal subject for studying fruit degreening and colouration processes (Yuan et al., 2015). Fruit colouration is influenced by a wide range of factors, with transcriptional regulation playing a crucial role in determining external appearance (Lu et al., 2024; Stanley and Yuan, 2019). To date, only a limited number of transcription factors (TFs) have been identified to regulate chlorophyll and carotenoid metabolism (Lu et al., 2024). It was found that *CcGCC1*, a MYB-related transcription factor, whose expression abnormally correlated with the delayed chlorophyll degradation in two *Citrus clementina* mutants (39B3 and 39E7) (Ríos et al., 2010). Meanwhile, there was a negative correlation between chlorophyll content and CitAP2/ERF family genes such as *CitERF5*, *CitERF6*, *CitERF7*, and *CitERF13* (Xie et al., 2014). Among them, two transcription factors, CitERF13 and CitERF6, were strongly associated with fruit degreening (Li et al., 2019; Xie et al., 2017; Yin et al., 2016). Additionally, in navel sweet orange, CsMADS3 has been shown to activate the promoter of *SGR*, a key gene involved in chlorophyll degradation, leading to decreased chlorophyll levels, and enhance carotenoid biosynthesis by activating the promoters of *PSY1* and *LCYB2* (Zhu et al., 2023).

The molecular regulatory mechanisms of carotenoids in citrus remain poorly understood, with few transcription factors being associated with carotenoid metabolism. Of note, overexpression of *CubHLH1*, identified in *Satsuma mandarin* (*Citrus unshiu* Marc.), in tomatoes reduced lycopene levels and altered the expression of carotenoid biosynthesis genes (Endo et al., 2016). On the other hand, in Green Ougan (MT), a spontaneous stay-green mutant of the commercial variety Ougan (WT) (*Citrus reticulata* cv *Suavissima*), CrMYB68 negatively regulates *BCH2* and *NCED5*, thereby suppressing α- and β-carotene transformation. While also interacting with CrNAC036 to synergistically suppress *NCED5* expression and abscisic acid (ABA) biosynthesis (Zhu et al., 2017, 2020). In addition, CsPHL3 directly suppresses *PSY* transcription, leading to decreased carotenoid content in ‘Anliu’ sweet orange (Lu et al., 2021a). Meanwhile, CsMADS6 forms a transcriptional complex with CsMADS5, which activates the transcription of *PSY*, *PDS*, and *LCYB1* by binding to their promoters, thus promoting carotenoid biosynthesis in ‘Hong Anliu’ sweet orange (*Citrus sinensis*) (Lu et al., 2021b, 2018). CsERF061 positively regulates *LCYB2*, and nine other genes involved in the carotenoid biosynthesis pathway, thereby enhancing carotenoid accumulation and increasing chromoplast numbers in navel orange (*Citrus sinensis*) (Zhu et al., 2021). FcrNAC22, which is induced by red light, directly binds to and activates the promoters of *LCYB1*, *BCH2*, and *NCED5*, facilitating carotenoid accumulation in kumquat fruit (Gong et al., 2021). Methyl jasmonate activates the CsMPK6–CsMYC2 signaling cascade, with CsMYC2 binding to the *CCD4b* promoter to enhance β-citraurin production, thereby intensifying the redness of ‘Newhall’ orange (*Citrus sinensis*) (Yue et al., 2023). A study showed that CsTT8 promotes fruit coloration through by controlling methylerythritol 4-phosphate (MEP) pathway and carotenoid synthesis in ‘Valencia’ orange (*Citrus natsudaidai*) (Sun et al., 2023a). CsHB5 and CsbZIP44 precisely modulate ABA signal-mediated carotenoid metabolism in ‘Valencia’ orange (*Citrus sinensis*) (Sun et al., 2023b). A recent study demonstrated that the transcriptional regulatory module CsERF110–CsERF53 orchestrates the coloration of citrus fruits in response to ABA signaling in ‘Valencia’ orange (Sun et al., 2024a). Furthermore, in *Satsuma mandarin* (*Citrus unshiu* Marc.), CitZAT4 directly binds to the promoters of *LCYB*, *HYD*, and *NCED2*, regulating their expression and promoting the accumulation of β-branch orange carotenoids (Sun et al., 2024b).

Although the biosynthetic pathways of chlorophyll and carotenoids have been extensively studied in various plants, including citrus fruits, the underlying transcriptional regulatory mechanisms remain less well understood. Previous researches have demonstrated that gibberellic acid and ethylene play significant roles in regulating fruit peel coloration (Alós et al., 2006; Gambetta et al., 2014; Pech et al., 2012; Wang et al., 2022; Yu and Wang, 2020). In recent years, multi-omics approaches have increasingly been employed to identify key regulatory genes associated with metabolites (Huang et al., 2024; Liu et al., 2023; Wu et al., 2022). Consequently, this study performed integrated transcriptomic and metabolomic analyses on citrus peels treated with GA and ETH to study the molecular mechanism of pigment metabolism. Comprehensive phenotypic, metabolomic, and transcriptomic analyses revealed extensive gene–metabolite regulatory networks that govern citrus peel coloration. These insights provide a valuable resource for advancing high-throughput research in citrus.

## Materials and methods

### Plant materials and sample collection

‘Murcot’ tangor (*Citrus reticulata* × *Citrus sinensis*) grafted on ‘Trifoliate orange’ (*P. trifoliata* L. Raf.) was used in this study. The trees were seven years old at the time of sampling, with a planting density of 3 m × 4 m. The experimental orchard was located in the Jiangjin District, Chongqing, China (116° 34′ E, 36° 50′ N), an area that experiences a warm, humid monsoon climate. Nine trees with uniform growth and similar fruit-bearing capacity were selected for this study. At 210 days post-anthesis (DPA), corresponding to the developmental stage where citrus fruits have attained approximately 90% of their final size (BBCH-scale: 79), the fruits were evenly sprayed with ultrapure water (CK), GA (100 ppm), and ETH (200 ppm). Citrus peels were collected at 15, 45, and 75 days post-treatment (DPT), resulting in sample names CK15, CK45, CK75, GA15, GA45, GA75, ETH15, ETH45, and ETH75. Five fruits were collected from each tree across five distinct (east, south, central, north, and west), and a total of 15 fruits, obtained from three trees, constituted a single biological replicate. Peels, which included just the exocarp, were immediately frozen in liquid nitrogen, transported on dry ice, and stored at –80°C for subsequent analysis. Additionally, 54 citrus fruits were collected for phenotype validation and chlorophyll determination.

### Metabolite identification and quantification

Samples were ground into a powder, and 50 mg was weighed and extracted using 0.5 mL of a mixed solution of n-hexane, acetone, and ethanol (1:1:1, v/v/v). The extract was vortexed for 20 minutes at room temperature, and the supernatant was collected after centrifugation at 12,000 rpm for 5 minutes at 4°C. The residue was then re-extracted using the same method. The combined extracts were evaporated to dryness and reconstituted in a MeOH/MTBE (methanol/methyl tert-butyl ether, 1:1, v/v) solution. The resulting solution was filtered through a 0.22 μm membrane for further LC-MS/MS analysis (Amorim-Carrilho et al., 2014).

Sample extracts were analyzed using a UPLC–APCI–MS/MS system (UPLC, ExionLC™ AD; MS, Applied Biosystems 6500 Triple Quadrupole). The analytical conditions were as follows: Column, YMC C30 (3 μm, 100 mm × 2.0 mm i.d.); Solvent system, methanol (1:3, v/v) with 0.01% butylated hydroxytoluene (BHT) and 0.1% formic acid (A) and MTBE with 0.01% BHT (B). The gradient program began at 0% B (0–3 min), increased to 70% B (3–5 min), then raised to 95% B (5–9 min), and returned to 0% B (10–11 min). The flow rate was set to 0.8 mL/min, with a column temperature of 28°C and an injection volume of 2 μL (Geyer et al., 2004). Mass spectrometric data were acquired using a triple quadrupole–linear ion trap mass spectrometer (QTRAP^®^ 6500+ LC–MS/MS System) equipped with an APCI Heated Nebulizer, operated in positive ion mode, and controlled via Analyst 1.6.3 software. The APCI source settings were as follows: ion source APCI+; source temperature, 350°C; curtain gas, 25.0 psi. Carotenoids were analyzed using scheduled multiple reaction monitoring (MRM). Data acquisition and metabolite quantification were performed using Analyst 1.6.3 and MultiQuant 3.0.3 software (Sciex). Mass spectrometer parameters, such as declustering potentials (DP) and collision energies (CE), were optimized for each MRM transition (Geyer et al., 2004; Krinsky et al., 2004).

### RNA sequencing analysis

Total RNA was extracted from citrus peels (in triplicate) using TRIzol^®^ Reagent following the manufacturer’s instructions (Invitrogen), with genomic DNA removed using DNase I (TaKara). RNA quality was assessed using an Agilent 2100 Bioanalyzer and quantified with a NanoDrop ND–2000 (NanoDrop Technologies). Only high-quality RNA samples (OD260/280 = 1.8–2.2, OD260/230 ≥ 2.0, RIN ≥ 6.5, 28S:18S ≥ 1.0, >1 μg) were used for library preparation.

RNA-seq transcriptomic libraries were prepared using the TruSeq™ RNA Sample Preparation Kit (Illumina), with 1 μg of total RNA as input. Messenger RNA was isolated using poly-A selection, fragmented, and then used to synthesize double-stranded cDNA with a SuperScript Double-Stranded cDNA Synthesis Kit (Invitrogen). Following end-repair and phosphorylation, the libraries were size-selected for cDNA fragments of 300 bp and PCR-amplified for 15 cycles. The libraries were quantified using TBS380 and sequenced on an Illumina NovaSeq 6000 system.

Raw paired-end reads were trimmed and quality-controlled using SeqPrep and Sickle with default parameters. Clean reads were aligned to the reference genome (*Citrus_clementina*_v1.0, GCF_000493195.1) using HISAT2 (Kim et al., 2015). Mapped reads were assembled using StringTie in a reference-based approach (Pertea et al., 2015). Differential expression analysis was conducted using DESeq2 (Love et al., 2014), with differentially expressed genes (DEGs) defined as |log2Foldchange| > 1 and Q-value ≤ 0.05 (Love et al., 2014).

### Hub gene identification using weighted gene co-expression network analysis

WGCNA was performed to identify hub genes (Fuller et al., 2007). Cluster analysis was based on gene expression (FPKM, fragments per kilobase million). A soft thresholding power of 18 was selected, as it was the minimum power that properly fit the scale-free topological index. The correlation between module eigengenes and traits was assessed, and hub genes were defined as having module membership (MM.abs) > 0.7 and gene significance (GS.abs) > 0.6. Gene interaction networks were visualized using Cytoscape, and transcription factor annotations were retrieved from PlantTFDB (Jinpu et al., 2017).

### Transient expression in tobacco epidermal cells

*Agrobacterium* cultures were resuspended in infiltration buffer (10 mM MgCl_2_ and 100 µM acetosyringone) to an OD600 of ~0.8 and then infiltrated into 8-week-old *Nicotiana benthamiana* leaves (Sparkes et al., 2006). After 5 days at 25°C, the infiltrated leaves were harvested for analysis.

### Chlorophyll determination

Chlorophyll content was measured according to a previous study (Porra et al., 1989). Chlorophyll was extracted thrice with 80% acetone and centrifuged at 12,000 g for 5 minutes at 4°C. The combined supernatant was adjusted to 10 mL with 80% acetone, and absorbance at 663 nm and 645 nm was measured.

### Statistics analysis

Principal component analysis (PCA) was conducted using RStudio (v2023.06.1–524) with the FactoMineR and factoextra packages (Lê et al., 2008; Team, 2008). The column stacking diagram was created in RStudio using the ggplot2 package. Heatmaps and clustering analysis were performed using TBtools (Chen et al., 2020). The column diagram was generated with GraphPad Prism 5. Heatmap & clustering map, and Venn diagram were analyzed using SRplot (https://www.bioinformatics.com.cn), an online platform for data analysis and visualization (Tang et al., 2023). The analysis results for polar column charts and bidirectional grouping bar chart plots were generated using the CNSknowall platform (https://cnsknowall.com a comprehensive web service for data analysis and visualization).

## Results

### GA reduces chlorophyll degradation, while ETH promotes this processes.

**Figure 1:**
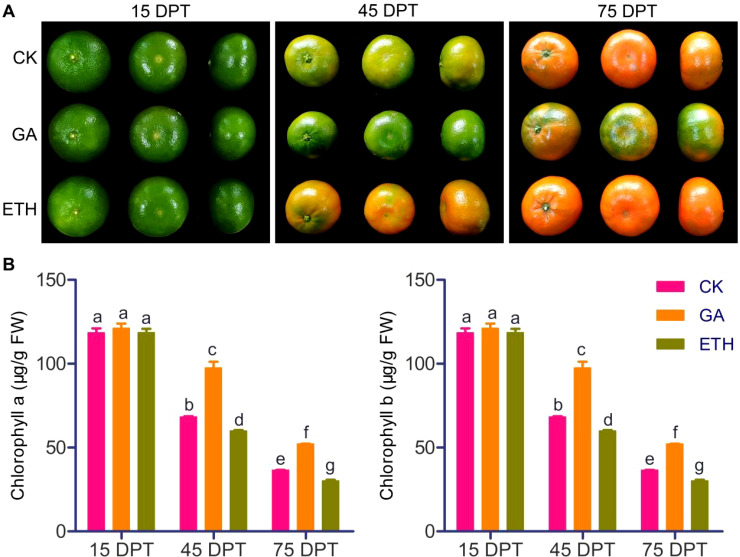
Effects of GA and ETH on citrus peel coloration and chlorophyll content at various developmental stages. **(A)** GA treatment delayed citrus peel coloration, while ETH treatment accelerated this process. **(B)** GA inhibited chlorophyll degradation in citrus peel, while ETH promoted chlorophyll degradation. Data are represented as mean ± standard deviation. FW refers to fresh weight. Different letters indicate statistically significant differences (*p* < 0.01).

The impact of GA and ETH on citrus peel coloration and chlorophyll content was assessed. There were no differences in peel color between GA, ETH, and control groups at 15 DPT. At 45 DPT, citrus peels treated with GA were still not showing coloration, but ETH-treated peels and control peels had already shown an orange color, with the ETH-treated peels being a more pronounced orange. At 75 DPT, the peels treated with CK and ETH were fully colored, whereas those treated with GA had not yet reached full coloration (**Figure 1A**). The chlorophyll content changes were associated with the phenotypic alterations. At 45 and 75 DPT, the chlorophyll content exhibited a hierarchical trend: GA > CK > ETH, despite no significant differences at 15 DPT (**Figure 1B**). These findings corroborate the known effect that GA delays peel coloration and reduces chlorophyll degradation, while ETH promotes these processes.

### Metabolomics analysis reveals differential carotenoid accumulation under GA and ETH treatment

**Figure 2:**
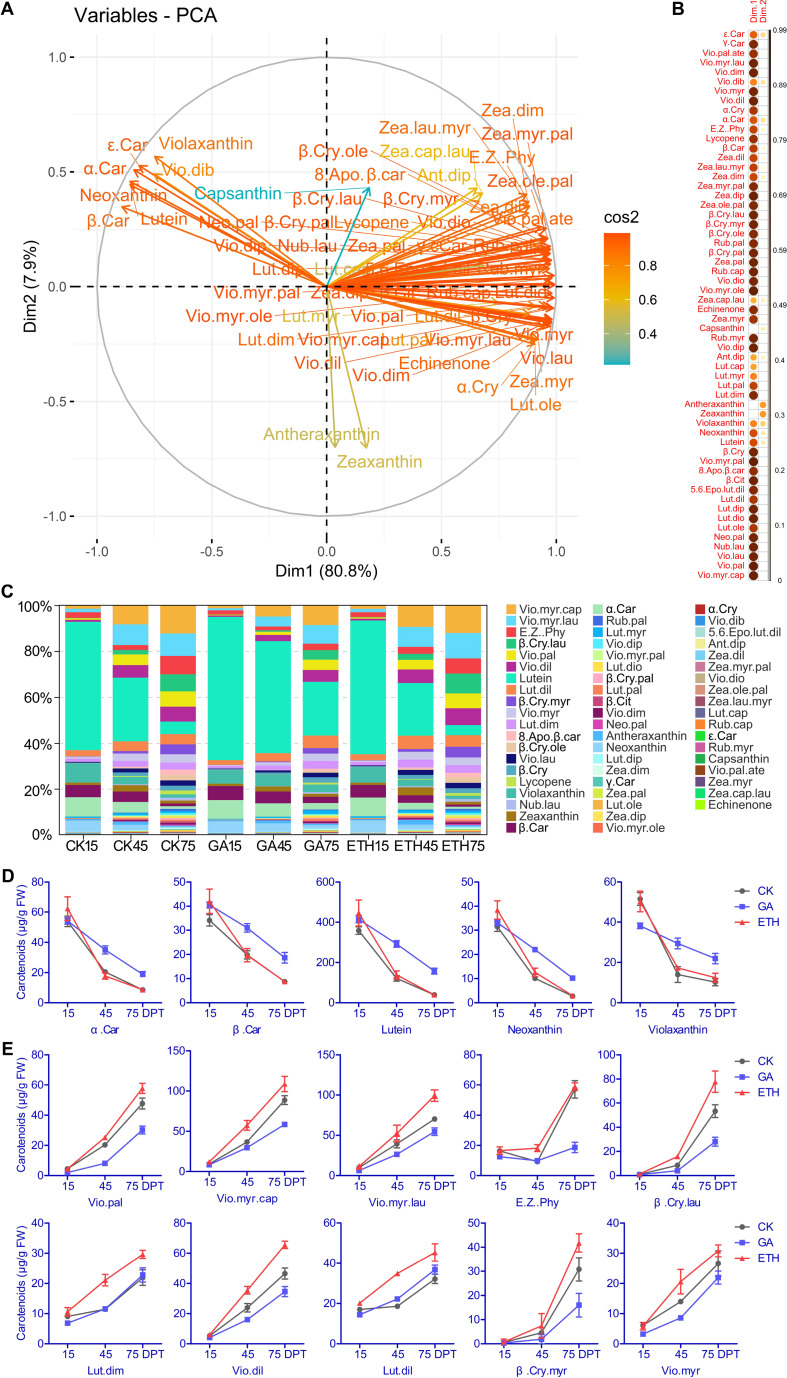
PCA analysis investigated the relationship between metabolites and citrus peel coloration and the changes in carotenoid proportions. **(A)** Variable correlation plots of 58 carotenoids, with colors representing the cos² values for PC1 and PC2. The distance of each variable from the origin reflects its contribution to the factor map. **(B)** Heatmap of cos² values for the variables in two dimensions. **(C)** Distribution of carotenoids across different treatments and time points. The top five carotenoids showing decreased levels **(D)**, and the top ten carotenoids exhibiting increased levels during development **(E)**. FW represents fresh weight. Abbreviations are provided in **Supplementary Table S1**.

Metabolomic analysis of 27 citrus samples identified 58 carotenoid-related metabolites (**Supplementary Table S1**). During the same period, the total carotenoid content in ETH was the highest. At 75 days, the ranking of total carotenoid content was ETH > CK > GA, suggesting that GA inhibits carotenoid synthesis, whereas ETH promotes this process (**Supplementary Table S1**). PCA analysis demonstrated that the first two principal components (PCs) accounted for 88.7% of the variance, effectively capturing the distribution of carotenoids (**Figure 2A**). Thirteen compounds, primarily colored carotenoids, exhibited strong associations with PC1 (variable correlation > 0.95) (**Figure 2B**; **Supplementary Table S2**). Seven carotenoids negatively correlated with PC1 exhibited and decline over time, whereas 51 carotenoids positively correlated with PC1 showed an increase over time (**Figure 2A**; **Supplementary Figures S1, S2**). A comparative analysis of the carotenoid compositions across three groups indicated that lutein was the predominant carotenoid at both 15 and 45 DPT. By 75 DPT, violaxanthin myristate–caprate emerged as the predominant carotenoid in the CK and ETH groups, while lutein continued to dominate in the GA group (**Figure 2C**; **Supplementary Table S1**). These findings confirm the known effect that lutein is the most abundant carotenoid in the immature pericarp and that GA inhibits the downregulation of lutein.

At 15 days, the top five carotenoids constituted 83-87% of the total carotenoid content, with their levels gradually declining during fruit development. GA was observed to inhibit this reduction (**Figure 2D**; **Supplementary Table S1**). By 75 days, the top ten carotenoids represented approximately 72% of the total content, with their levels progressively increasing as the fruit developed. Notably, the accumulation of five violaxanthin esters (violaxanthin palmitate, violaxanthin myristate–caprate, violaxanthin myristate–laurate, violaxanthin dilaurate, violaxanthin myristate) and two β-cryptoxanthin derivatives (β–cryptoxanthin laurate, β–cryptoxanthin myristate) was inhibited by GA but promoted by ETH (**Figure 2E**; **Supplementary Table S1**). These results indicated that hormonal regulation plays a significant role in the modulation of carotenoid levels during the development of citrus peel.

### An overview of transcriptomic and identification of transcription factors regulating pigment metabolism by DEGs analysis

**Figure 3:**
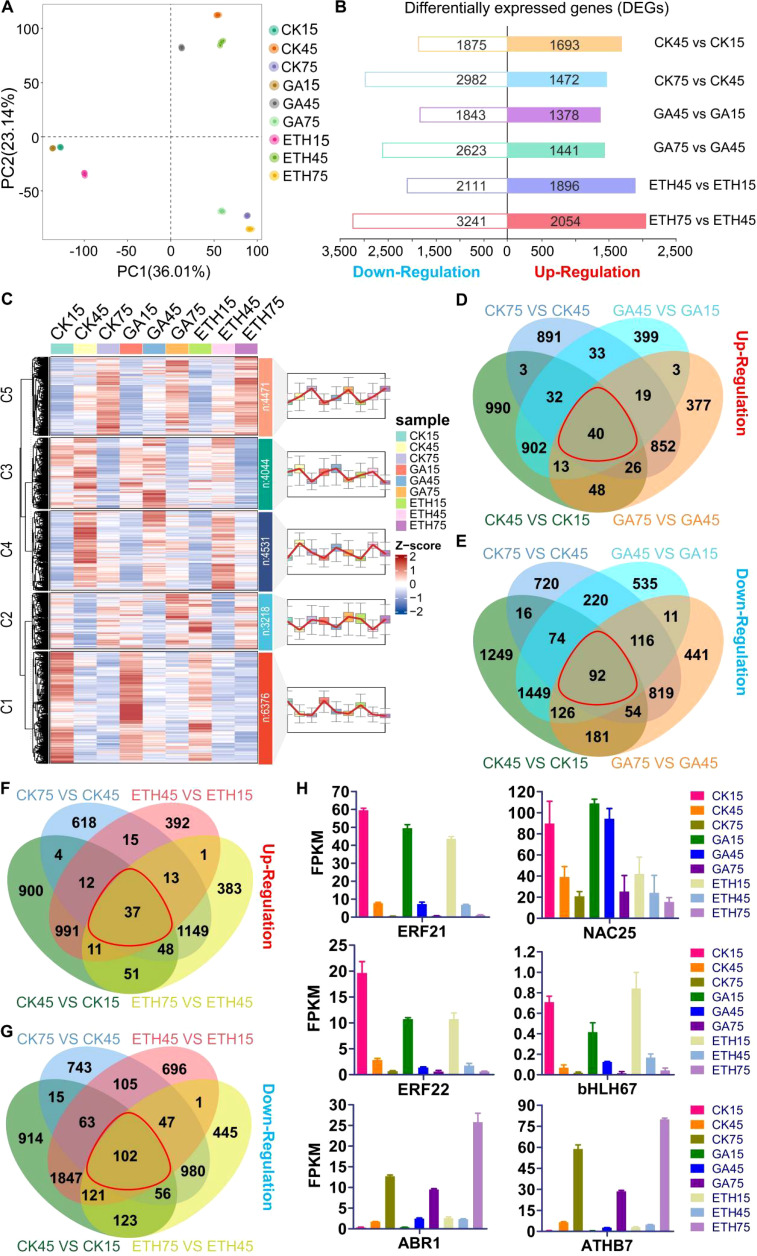
Variability of transcript levels in citrus peel under GA and ETH treatments. **(A)** PCA analysis. **(B)** DEGs count across various comparison groups. **(C)** Gene expression patterns are categorized into five clusters, labeled as C1 through C5, representing clusters 1 to 5, respectively. Here, ‘n’ denotes the number of genes in each cluster. **(D–G)** Venn diagrams depicting DEG counts across comparison groups, with each red line indicating the intersections among the four comparison groups. the control group displayed on the right side of each comparison. **(H)** Expression levels of six candidate transcription factor genes.

A total of 1.78 billion clean paired-end reads were obtained from the RNA-seq dataset for 27 samples, with a mapping rate ranging from 76.9% to 88.6% to the *Citrus clementina* genome (**Supplementary Tables S3, S4**). PCA analysis demonstrated that the first two principal components accounted for 59.2% of the variation, with three replicates of each sample clustering closely together, indicating high consistency and quality of the data. Hierarchical clustering analysis (HCA) and PCA results revealed that transcriptomic profiles were primarily separated by time points (**Figure 3A**; **Supplementary Figure S3**). A total of 11,972 DEGs, constituting 47.86% of the genome, were identified (**Supplementary Table S5**). The CK and ETH groups exhibited more DEGs than the GA group during development, aligning with the phenotypic observations of peel coloration (**Figure 3B**). Clustering analysis revealed five distinct gene expression patterns (**Supplementary Table S7**; **Supplementary Figure S4**). Genes in cluster 5 showed progressive upregulation during fruit development, while clusters 1 and 3 exhibited gradual downregulation (**Figure 3C**). Together, these three clusters contain 14,891 genes, accounting for 65.8% of the total gene set (**Supplementary Table S6**).

A venn analysis of upregulated genes across the comparison groups CK75 vs. CK45, CK45 vs. CK15, GA75 vs. GA45, and GA45 vs. GA15 identified 40 common genes (**Figure 3D**). Similarly, a venn analysis of downregulated genes within the same comparison groups revealed 92 common genes (**Figure 3E**). Furthermore, a venn analysis of upregulated genes in the comparison groups CK75 vs. CK45, CK45 vs. CK15, ETH75 vs. ETH45, and ETH45 vs. ETH15 identified 37 common genes (**Figure 3F**), while the analysis of downregulated genes in these groups revealed 102 common genes (**Figure 3G**). In total, 199 genes were identified through venn analysis, including six transcription factor genes (**Supplementary Table S7**). Among the six transcription factor genes, the expression levels of four genes (*ERF21*, *ERF22*, *bHLH67*, and *NAC25*) were observed to be downregulated, whereas two genes (*ABR1* and *ATHB7*) exhibited upregulation during development stages (**Figure 3H**). Furthermore, our findings indicate that the expression levels of four genes associated with carotenoid metabolism and five genes related to chlorophyll metabolism were inhibited by GA, yet promoted by ETH (**Supplementary Figure S5**).

### Gene screening using WGCNA

**Figure 4:**
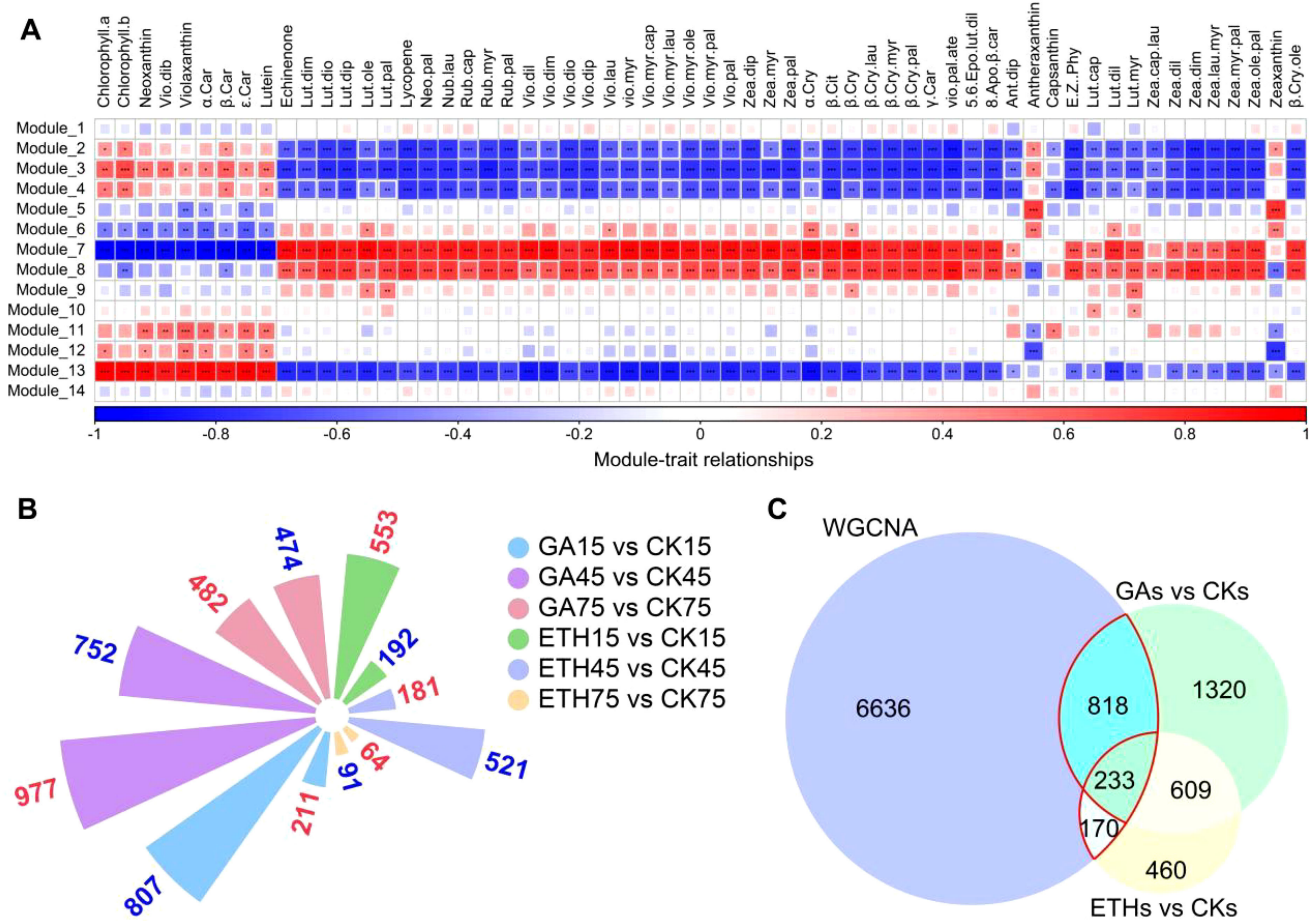
WGCNA analysis. **(A)** Heatmap illustrating the correlation between modules and pigments (carotenoids and chlorophyll). The gene significance (GS) value for each module–pigment pair is represented by color intensity and square size. Asterisks (*, **, ***) denote *p*-values less than 0.05, 0.01, and 0.001, respectively. Positive correlations are indicated in red, while negative correlations are shown in blue. **(B)** Bar graph depicting the number of DEGs that are either upregulated or downregulated across various comparison groups. Upregulated genes are displayed in red font, and downregulated genes in blue font. The control group for each comparison is represented on the right-hand side. **(C)** Venn diagrams illustrating the overlap between differentially expressed genes across comparison groups and hub genes derived from WGCNA modules 7, 8, and 13. “GAs vs. CKs” refers to the collection of GA15 vs. CK15, GA45 vs. CK45, and GA75 vs. CK75, while “ETHs vs. CKs” refers to ETH15 vs. CK15, ETH45 vs. CK45, and ETH75 vs. CK75. The intersections marked by red lines indicate the final candidate genes.

Based on transcriptomic and metabolomic data, a weighted correlation network was constructed using 25,017 transcripts for identifying co-expression modules and hub genes. A soft thresholding power of 18 was selected, and 14 modules were revealed after the merged dynamic analysis (**Figure 4A**; **Supplementary Figure S6**). Most genes (5,223) were categorized into Module 13, while five other modules (Modules 2, 5, 7, 8, and 14) contained between 1,027 and 3,884 genes. The remaining eight modules comprised between 77 and 610 genes (**Supplementary Table S8**).

A wide range of correlation coefficients was found between module content and pigment (chlorophyll and carotenoids) content, from -0.98 to 95 (**Supplementary Table S9**). Of note, three modules (Modules 7, 8, and 13) exhibited GS-values exceeding 0.6 across multiple compounds, indicating a marked correlation between the genes in these modules and pigment content (**Figure 4A**; **Supplementary Table S9**). A total of 7,857 hub genes were identified based on the criteria of |MM| > 0.8 and |GS| > 0.7 (**Supplementary Table S11**).

We identified DEGs across the following comparisons: GA15 vs. CK15, GA45 vs. CK45, GA75 vs. CK75, ETH15 vs. CK15, ETH45 vs. CK45, and ETH75 vs. CK75 (**Figure 4B**; **Supplementary Table S5**). These DEGs were grouped into two collections: GAs vs. CKs (GA15 vs. CK15, GA45 vs. CK45, GA75 vs. CK75) and ETHs vs. CKs (ETH15 vs. CK15, ETH45 vs. CK45, ETH75 vs. CK75). These differential gene sets (GAs vs. CKs, ETHs vs. CKs) were then subjected to venn analysis, along with the hub genes identified via WGCNA, leading to the identification of 1,221 candidate genes (**Figure 4C**; **Supplementary Table S11**).

### Network analysis and validation candidate genes associated with pigment metabolism

**Figure 5:**
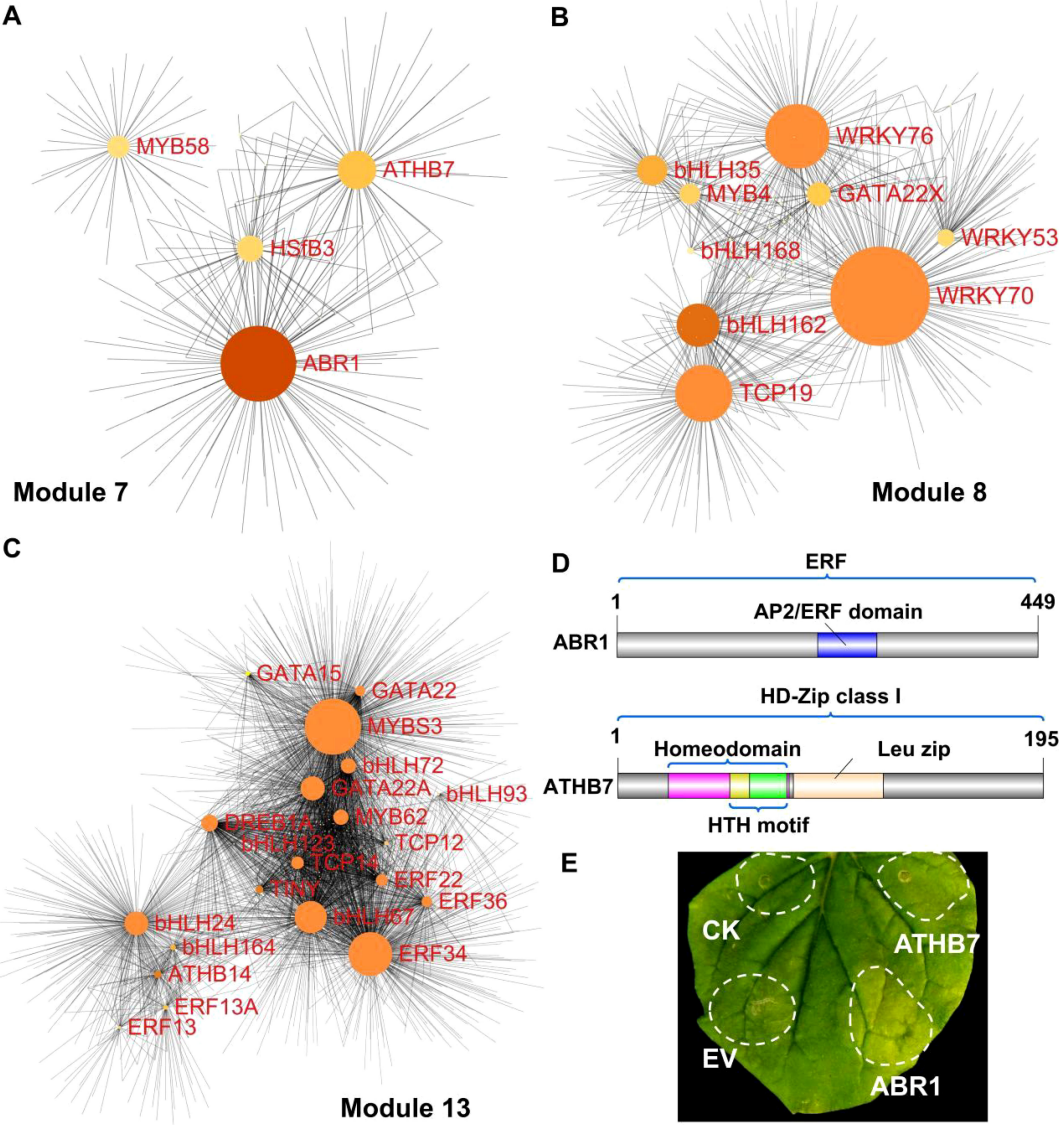
Correlation network of three modules and functional characterization of genes via transient expression in tobacco. **(A–C)** Correlation networks of module 7 **(A)**, module 8 **(B)**, and module 13 **(C)**. Node size reflects the number of connected genes. **(D)** Protein structure diagrams of ABR1 and ATHB7, showing the AP2/ERF domain of ABR1, and HTH (helix-turn-helix) motif and the leucine zipper domain of ATHB7 **(E)** Transient expression analysis of ABR1 and ATHB7 in tobacco leaves, with control (CK) indicating the infiltration buffer and EV representing the empty vector.

Based on the hub transcription factor (Hub–TF) genes and their correlation network, we constructed and visualized a network closely associated with carotenoid and chlorophyll metabolism, applying a weight threshold greater than 0.2 (**Figures 5A–C**; **Supplementary Table S12**). In total, 4, 9, and 20 transcription factors were identified in Modules 7, 8, and 13, respectively (**Figures 5A–C**; **Supplementary Table S12**).

Notably, two transcription factor genes, *ABR1* and *ATHB7*, which were also identified through DEGs analysis (**Figure 3H**), were selected for functional verification in the regulation of pigment metabolism (**Figures 3H**, **5A**). Protein domain analysis revealed that ABR1 is part of the ERF subfamily and possesses a single AP2/ERF domain, and ATHB7 belongs to the class I subfamily of HD-Zip, containing a homeodomain, a leucine zipper domain, and an HTH motif (**Figure 5D**). The expression of *ABR1* and *ATHB7* was progressively upregulated during fruit development, suggesting a potential positive role in the regulation of peel coloration. Transient overexpression of *ABR1* and *ATHB7* in tobacco leaves resulted in accelerated leaf degreening (**Figure 5E**), indicating that both ABR1 and ATHB7 may play crucial roles in regulating pigment metabolism. The results of the Tobacco color index (CI) analysis demonstrated that the CI values for leaves overexpressing *ABR1* and *ATHB7* were higher compared to the control groups (CK and EV), indicating a shift in leaf coloration from green to yellow (**Supplementary Figure S7**).

## Discussion

Globally, citrus contributes substantial value to various countries and regions due to its economic impact (Mahato et al., 2018). Fruit color is a critical aesthetic quality that directly impacts consumer preference for citrus products (Ruiz-Sola and Rodríguez-Concepción, 2012; Yuan et al., 2015). Although the roles of gibberellic acid and ethylene in fruit ripening have been extensively investigated, their underlying regulatory mechanisms remain to be fully elucidated (Lu et al., 2024). Therefore, the present study evaluated the impact of GA and ETH treatments on citrus peel coloration and chlorophyll content at multiple time points (15, 45, and 75 DPT). Our results confirmed that GA delays the coloration of peels by inhibiting chlorophyll degradation, whereas ethylene facilitates it by accelerating chlorophyll degradation (**Figure 1**), consistent with previous research.

Citrus fruits contain over 100 carotenoid metabolites, with their specific content and composition significantly influencing the diverse coloration observed among different varieties (Rouseff et al., 1996). Accordingly, targeted metabolomics was employed to determine the impact of GA and ETH treatment on carotenoid content and composition (**Figure 2**; **Supplementary Table S1**). Metabolomics analysis result indicated a temporal decrease in seven carotenoids, whereas 51 carotenoids increased over time (**Figure 2**; **Supplementary Figures S1, S2**). Consistent with previous studies (Gambetta et al., 2014; Keawmanee et al., 2022; Yu and Wang, 2020), our study demonstrated that applying exogenous gibberellic acid before color break postpones color development by reducing carotenoid concentration, and altering carotenoid composition (**Figure 2**; **Supplementary Table S1**). Previous studies have shown that gibberellic acid delays the downregulation from the β, ϵ–branch to the β, β–branch, thereby maintaining higher lutein levels in the peel of *Clemenules mandarins* and navel oranges, while inhibiting the accumulation of phytoene and downstream xanthophylls such as β–cryptoxanthin, all–trans–violaxanthin, and 9–cis–violaxanthin (Alós et al., 2006; Keawmanee et al., 2022; Rodrigo and Zacarias, 2007). Herein, by 75 days, violaxanthin myristate–caprate (vio–myr–cap) emerged as the predominant carotenoid in both the CK and ETH groups, while lutein remained dominant in the GA group (**Figure 2C**; **Supplementary Table S1**). These findings suggest that GA inhibits the downregulation of lutein. Additionally, the accumulation of vio.pal, vio.myr.cap, vio.myr.lau, vio.dil, vio.myr β.cry.lau, and β.cry.myr, was inhibited by GA, while ETH promoted their accumulation (**Figures 2D, E**). GA also suppressed the downregulation of α-carotene, β-carotene, lutein, neoxanthin, and violaxanthin, while facilitating the accumulation of (E/Z)-phytoene (E.Z.phy). In contrast, ETH enhanced the accumulation of lutein dimyristate (lut.dim) (**Figures 2D, E**). Previous studies have demonstrated that ethylene increases total carotenoid content and elevates specific carotenoids such as β-cryptoxanthin, β-citraurin, and phytoene (Fujii et al., 2007; Rodrigo and Zacarias, 2007; Sun et al., 2024b). In line with these findings, our results also show that ethylene increased the total carotenoid content (**Supplementary Figure S1**; **Supplementary Table S1**) and the concentration of several individual carotenoids (**Figure 2**; **Supplementary Table S1**). Metabolomic analyses highlighted a key stage of hormone-driven carotenoid changes in citrus peel.

While the biosynthetic pathways of chlorophyll and carotenoids, along with the associated carotenogenic genes in citrus, have been characterized, knowledge of the regulatory networks involved remains limited (Concepcion et al., 2018; Lu et al., 2024). Transcriptomics has become essential for investigating the regulatory mechanisms underlying pigment metabolism (Huang et al., 2024; Wu et al., 2022; Zhang et al., 2021). In our research, transcriptomics analysis identified six transcription factors that were either upregulated or downregulated during maturation, including four types of TFs (ERF, NAC, bHLH, HD-zip), which has significant implications for pigment metabolism. Specifically, two genes, *ABR1* and *ATHB7*, were upregulated, while the remaining four genes were downregulated throughout development (**Figure 3**; **Supplementary Table S4**). This is consistent with previous studies, the two types of TFs are involved in regulating pigment metabolism (Sun et al., 2023b, 2024a).

In recent years, transcriptomic and metabolomic technologies have proven to be effective tools for elucidating the regulation of fruit metabolism during citrus development (Huang et al., 2024; Wu et al., 2022; Zhang et al., 2021). Herein, 33 transcription factors were identified through an integrative approach that combined transcriptomic and metabolomic analyses (**Figures 4**, **5A–C**). Previous research has shown that the regulatory roles of these transcription factors can be validated through transient overexpression of target genes in tobacco (Gong et al., 2021; Li et al., 2019; Yin et al., 2016; Zhu et al., 2021). Our findings demonstrated that the transient expression of *ABR1* and *ATHB7*, identified through both DEGs analysis and WGCNA, could promote the degreening of tobacco leaves (**Figures 3H**, **5E**), suggesting that ABR1 and ATHB7 may play critical roles in chlorophyll and carotenoids metabolism during fruit ripening, with their expression levels being modulated by GA and ETH.

## Conclusion

In summary, we obtained large-scale information on gene-metabolite regulatory networks related to peel coloration from comprehensive phenotypic, transcriptomic, and metabolomic analyses. GA inhibited the accumulation of five violaxanthin esters and two β-cryptoxanthin derivatives, while ETH promoted their accumulation. Moreover, GA suppressed the downregulation of lutein, the main carotenoid in immature fruits. Notably, two novel transcription factors, ABR1 and ATHB7, were found to regulate the coloration process by integrated transcriptomics and metabolomics analysis, as confirmed by transient overexpression assays in tobacco. Our results highlighted global transcriptional changes in citrus peel color regulation under different hormone conditions, which could be beneficial for citrus breeding.

## Data Availability

The datasets presented in this study can be found in online repositories. The names of the repository/repositories and accession number(s) can be found in the article/**Supplementary Material**.
