# Supplementary material for: New insights into the transcription factor regulatory networks driving peel coloration under hormone induction analyzed by transcriptomics and metabolomics in tangor ‘Murcot’
Source: Front Plant Sci. 2025 Feb 18;16:1526733. doi: 10.3389/fpls.2025.1526733 (PMC11876184; doi:10.3389/fpls.2025.1526733)
Supplement: Supplementary file 1 [file DataSheet1.zip › Supplementary materials/Supplementary materials (2).docx]

**Supplementary materials**

**
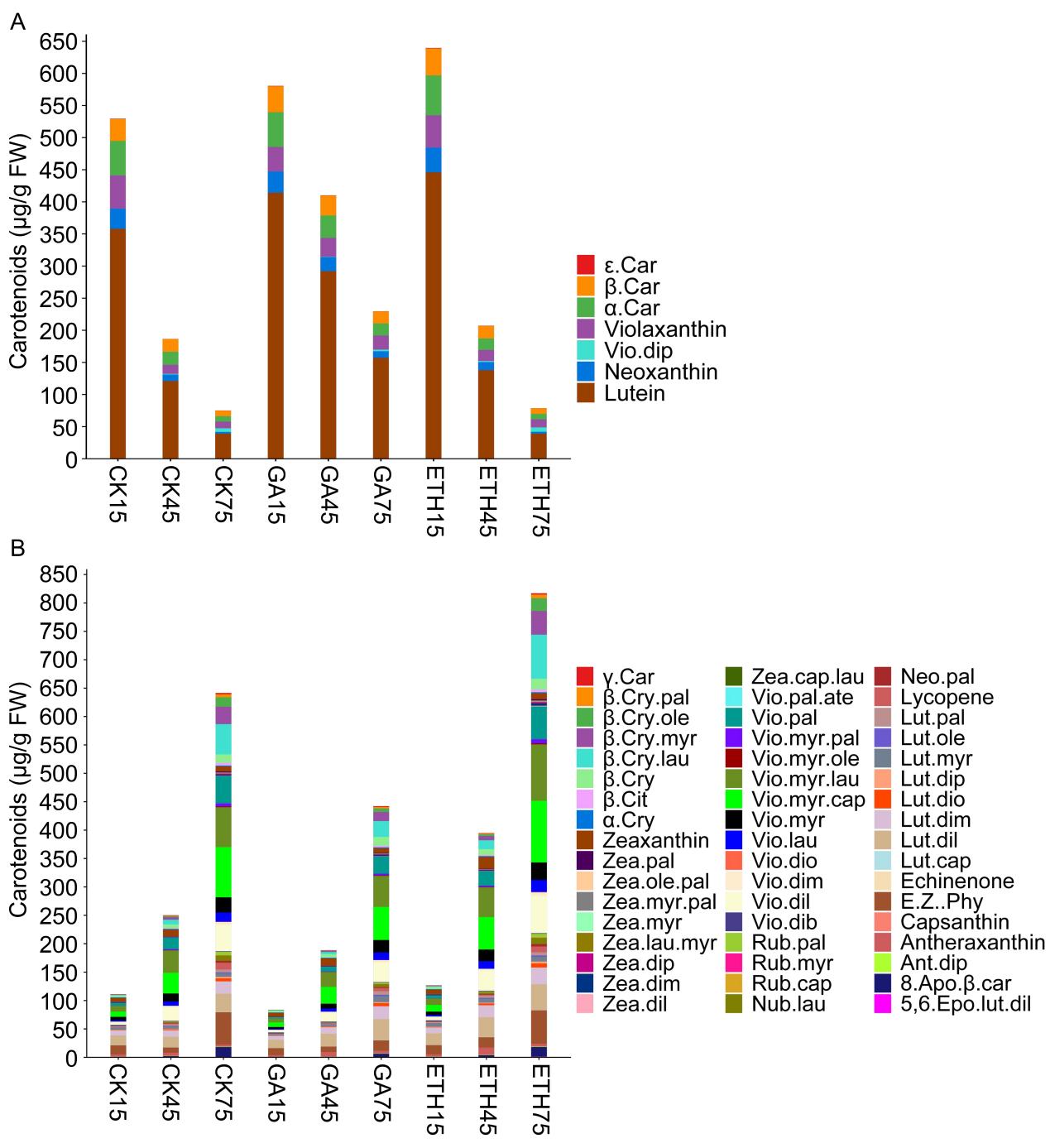
Figure S1:** Content of carotenoids. **(A)** Seven carotenoids negative associated with peel color. **(B)** Fifty-one carotenoids positeve associated with peel color. FW, fresh weight.


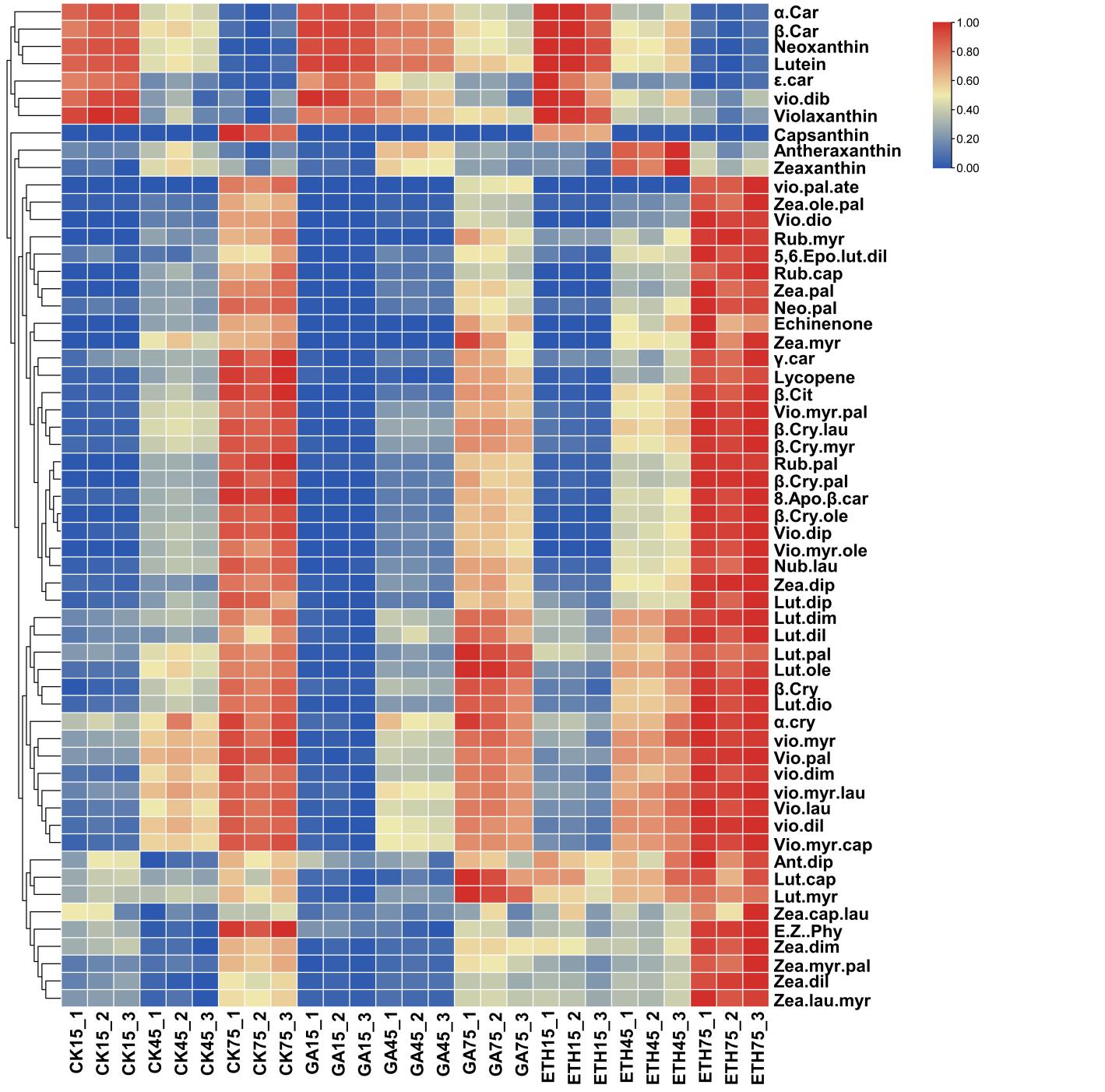


**Figure S2:** Cluster heat map of all carotenoids.


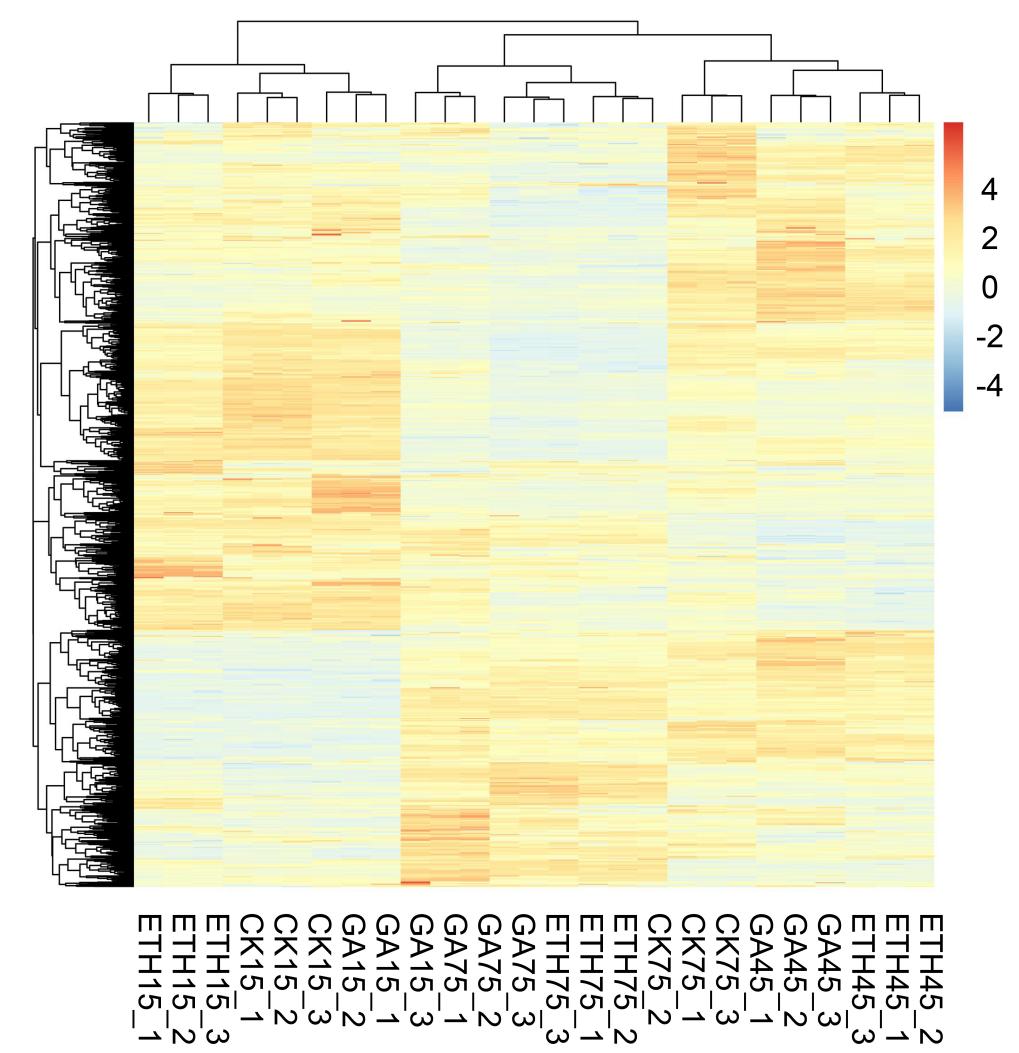


**Figure S3:** HCA of all genes from transcriptome data.


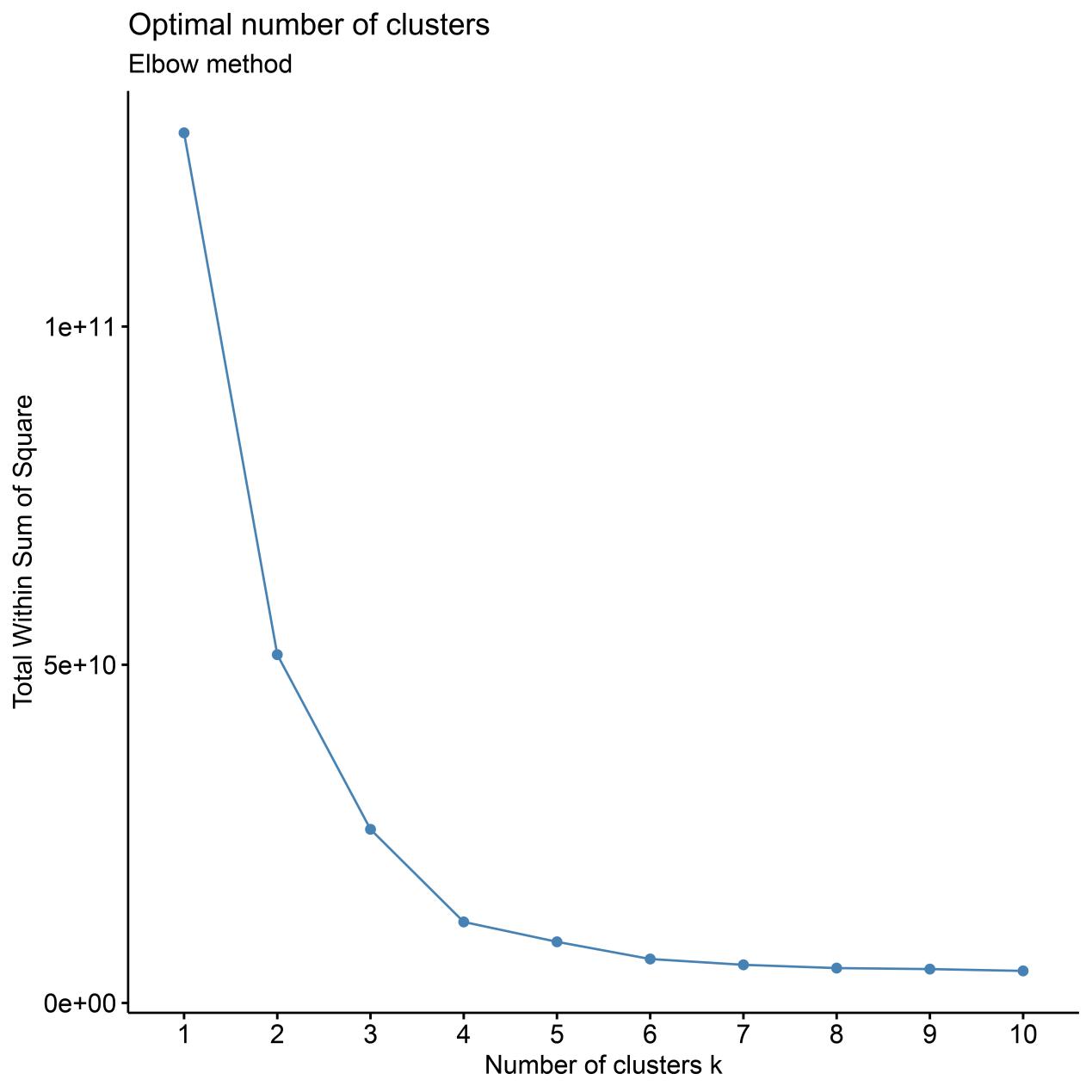


**Figure S4:** Optimal number of clusters.


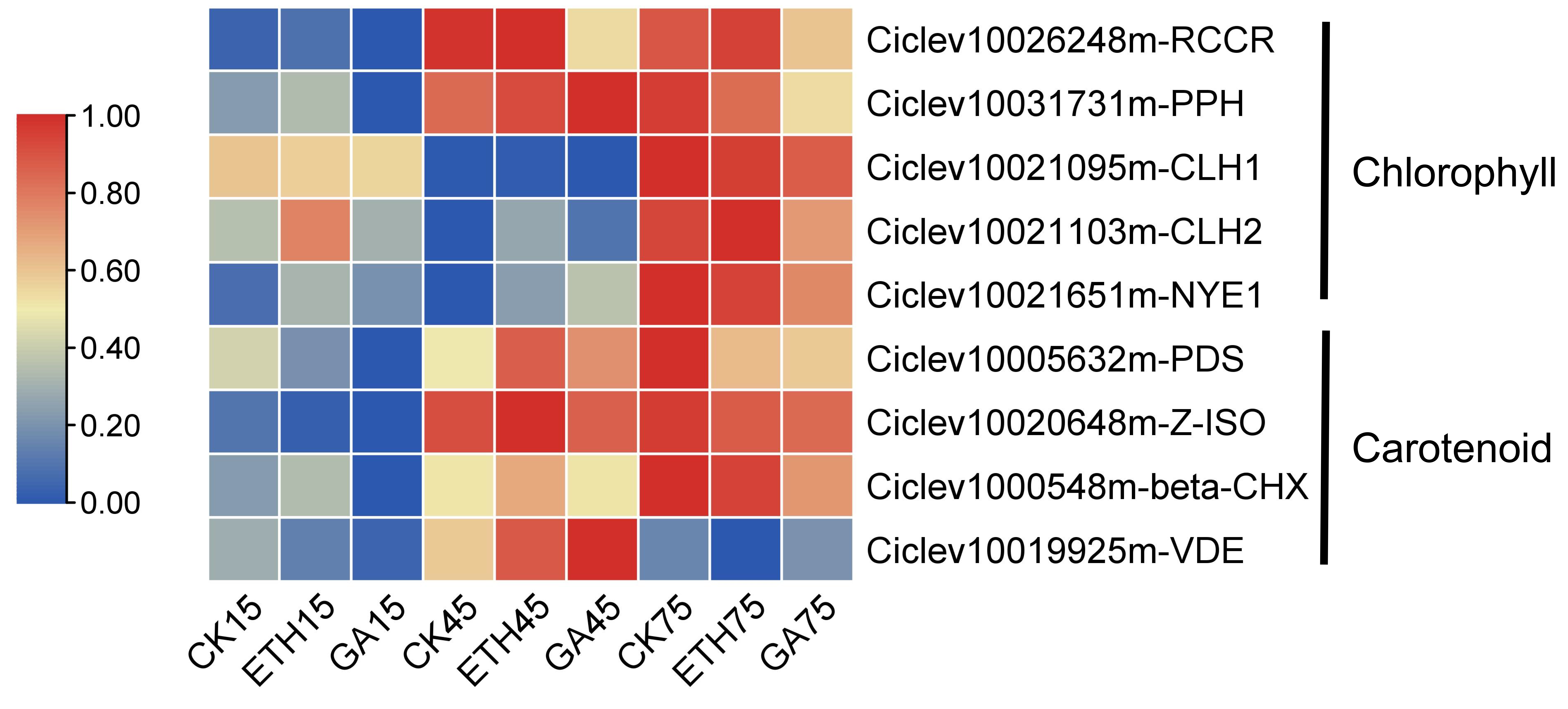


**Figure S5:** Heatmap of the expression level of carotenoid and chlorophyll metabolism related genes. RCCR, red Chl catabolite reductase; PPH, Pheophytinase; CLH, chlorophyllase; NYE, Non-Yellowing; PDS, phytoene desaturase; Z-ISO, 15-cis-zeta-carotene isomerase; β-CHX, beta-carotene hydroxylase; VDE, violaxanthin de-epoxidase.


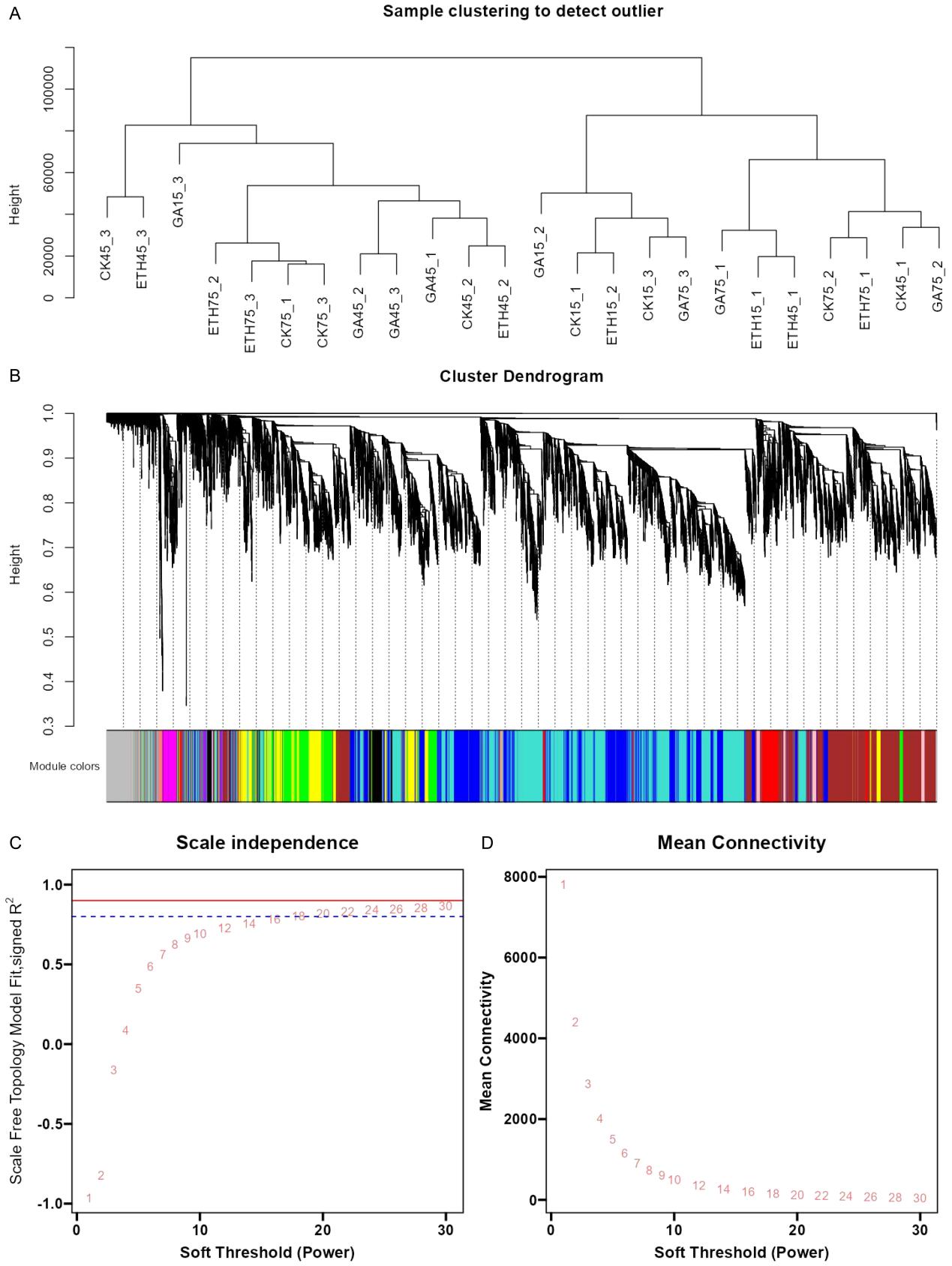


**Figure S6:** Weighted-gene co-expression network. **(A)** Sample clustering. **(B)** Hierarchical cluster dendrogram constructed by WGCNA, on which each leaf represents a gene. 14 merged modules (based on a threshold of 0.20) identified by weighted-gene co-expression network. **(C)** Scale free topology model fit. The dashed line represents R^2^ = 0.8. (D) Mean connectivity.


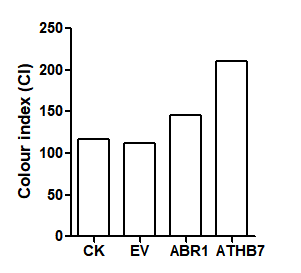


**Figure S7:** Changes in tobacco leaf colour index (CI). Increase in CI indicates the leaf colour changing from green to yellow.
